# Host RNA-Binding Proteins as Regulators of HIV-1 Replication

**DOI:** 10.3390/v17010043

**Published:** 2024-12-31

**Authors:** Sebastian Giraldo-Ocampo, Fernando Valiente-Echeverría, Ricardo Soto-Rifo

**Affiliations:** 1 Laboratory of Molecular and Cellular Virology, Institute of Biomedical Sciences, Faculty of Medicine, Universidad de Chile, Santiago 8380453, Chile; sebastian.giraldo@ug.uchile.cl (S.G.-O.); fvaliente@uchile.cl (F.V.-E.); 2 Center for HIV/AIDS Integral Research (CHAIR), Faculty of Medicine, Universidad de Chile, Santiago 8380453, Chile; 3 Millennium Institute in Immunology and Immunotherapy, Santiago 8380453, Chile

**Keywords:** HIV-1, RNA-binding proteins, RBPome, RNA metabolism

## Abstract

RNA-binding proteins (RBPs) are cellular factors involved in every step of RNA metabolism. During HIV-1 infection, these proteins are key players in the fine-tuning of viral and host cellular and molecular pathways, including (but not limited to) viral entry, transcription, splicing, RNA modification, translation, decay, assembly, and packaging, as well as the modulation of the antiviral response. Targeted studies have been of paramount importance in identifying and understanding the role of RNA-binding proteins that bind to HIV-1 RNAs. However, novel approaches aimed at identifying all the proteins bound to specific RNAs (RBPome), such as RNA interactome capture, have also contributed to expanding our understanding of the HIV-1 replication cycle, allowing the identification of RBPs with functions not only in viral RNA metabolism but also in cellular metabolism. Strikingly, several of the RBPs found through interactome capture are not canonical RBPs, meaning that they do not have conventional RNA-binding domains and are therefore not readily predicted as being RBPs. Further studies on the different cellular targets of HIV-1, such as subtypes of T cells or myeloid cells, or on the context (active replication versus reactivation from latency) are needed to fully elucidate the host RBPome bound to the viral RNA, which will allow researchers and clinicians to discover new therapeutic targets during active replication and provirus reactivation from latency.

## 1. Introduction

The replication cycle of human immunodeficiency virus type-1 (HIV-1) consists of two major phases: (i) the early phase, which begins when the viral particle attaches to the target cell and fuses its envelope with the plasma membrane, allowing the entry of the viral capsid, and finishes with the reverse transcription of the RNA genome into double-stranded DNA, which is integrated into host chromosomes, leading to a state known as “provirus”; and (ii) the late phase, in which the provirus is transcribed, viral proteins are synthesized, and new viral particles are assembled and released [1]. HIV-1 has a particular tropism for cells of the immune system that express the receptor CD4 [2] together with CCR5 or CXCR4, which the virus uses as co-receptors for cell attachment and entry [3], thus allowing the infection of CD4^+^ T cells but also other immune cell populations such as dendritic cells, monocytes/macrophages, and microglia [4]. In a subpopulation of infected cells such as memory CD4^+^ T cells, the virus establishes latency, a state characterized by the absence of proviral DNA transcription and viral particle production [5]. Latently infected cells (latent reservoir) can harbor the virus for decades and thus represent a major barrier to cure strategies [6].

Transcription from the proviral DNA gives rise to a 9 kb full-length RNA, which undergoes alternative splicing to allow the synthesis of more than 100 viral mRNAs that give rise to 15 viral proteins [7]. At the beginning of the late phase, the 9 kb RNA is fully/multiply spliced (MS RNA) into viral transcripts of about 2 kb, which code the regulatory proteins Tat and Rev and the accessory protein Nef. While the Tat protein is imported into the nucleus to transactivate viral transcription, the Rev protein is also imported into the nucleus to allow the accumulation and nuclear export of the 4 kb partially spliced RNA (PS RNA) that encodes the viral proteins Env, Vpu, Vif, and Vpr, and the unspliced full-length RNA (US RNA) that, once it has reached the cytosol, will be used as a genome incorporated into newly assembled virions or as the mRNA to produce the major structural polyprotein Gag (composed of the domains matrix (MA), capsid (CA) and nucleocapsid (NC), and p6) and Gag-Pol, which allows the synthesis of viral enzymes, protease (PR), integrase (IN), and reverse transcriptase (RT) [8,9].

Since the 15 viral proteins encoded in the HIV-1 genome are not sufficient to accomplish all the biological processes required for a successful replication cycle, the virus exploits many different cellular components and pathways through the interaction and usurpation of host proteins, which are recruited to both viral proteins and RNA [10]. In the former case, several host proteins have been shown to interact with viral proteins such as Env (e.g., HLA-E and molecules of the Notch1 signaling pathway) [11], Vif (e.g., host proteins involved in the control of nuclear transcription and nucleoside biosynthesis and proteins associated with the proteasome) [11], and Rev (which interacts with more than 100 host proteins) [12]. Accordingly, all HIV-1 proteins interact with several cellular proteins. Up to 435 proteins have been found to interact with at least one viral protein in Jurkat cells (a CD4^+^ T-cell model) and HEK293 cells (a widely used permissive cellular model) [13]. Strikingly, both cell lines only share nearly 40% of these viral protein–host protein interactions, suggesting that the cellular model for studying HIV-1 is significant.

On the other hand, less is known about the host proteins that are in direct interaction with the HIV-1 transcripts. Recent studies have demonstrated that hundreds of host proteins interact with the three types of viral transcripts (US, PS, and MS). Several approaches have been used to study the involvement of these RNA-binding proteins (RBPs) in HIV-1 infection. Classical strategies involve targeted studies in which selected proteins are depleted by genetic silencing (siRNA or CRISPR/Cas9) or overexpressed by the transfection of a plasmid or transduction with a viral vector encoding the desired protein. After treatment, cells are infected, and protein involvement in replication is assessed. If the protein affects viral replication, the underlying mechanism is studied, and immunoprecipitation, CLIP-qPCR, or other techniques are usually used to evaluate whether the protein interacts with the viral RNA and the implications of these interactions. However, in the past few years, strategies aimed at identifying the whole RBP interactome (RBPome) bound to HIV-1 transcripts by proteomic analysis have been developed. The identified proteins are then targeted as previously described, and their role in the replication cycle is evaluated. Nevertheless, the road to understanding the HIV-1 RBPome and its influence on viral replication still requires further investigation. This review focuses on the role of RNA-binding proteins (RBPs) that influence the fate of viral transcripts during HIV-1 replication through direct RNA-RBP interaction.

## 2. RNA-Binding Proteins as Critical Regulators of mRNA Fate

Cellular RNAs are always associated with proteins forming ribonucleoprotein (RNP) complexes. The proteins that are in direct contact with the RNA are called RNA-binding proteins (RBPs) and are active players in every step of RNA metabolism from birth to death, as they regulate transcription, splicing, modification, nuclear export, translation (in the case of mRNA), function, localization, and decay [14]. Therefore, RBPs add a complex layer of mRNA regulation that links RNAs to metabolic and other cellular pathways in extensive regulatory networks [15]. In humans, more than 3000 potential or candidate RBPs have been reported [15], which can be divided into two groups: “housekeeping” RBPs, which may be constitutively and ubiquitously active, and RBPs with more restricted and context-dependent expression [16]. Interestingly, the RBPome is not static, changing dynamically in response to environmental cues such as viral infections [17]. In addition, the expression of RBPs is context-dependent, as it can vary between cell types or be regulated by post-translational modifications or epitranscriptomic modifications of the RNA, among other processes [17]. As expected, viral RNAs also form RNP complexes with viral and cellular proteins [18,19,20].

## 3. RNA-Binding Proteins Identified by Targeted Studies as Regulators of HIV-1 Replication

HIV-1 encodes its own viral RNA-binding proteins (vRBPs), with Tat, Rev, and Gag (mainly through its NC domain) being the most important. Tat is one of the first viral proteins to be expressed by the virus during replication. Once produced, Tat interacts with the *p*-TEFb complex, composed of CycT1 and the cyclin-dependent kinase 9 (CDK9) in the cytoplasm, and then is imported into the nucleus, where it binds to the transactivating responsive (TAR) hairpin located at the very 5′ end of the nascent viral transcript, allowing the transcriptional elongation of the viral RNA by RNA polymerase ll and enhancing overall viral replication. Upon Tat depletion, the RNA polymerase ll is stalled in the TAR hairpin, dramatically limiting provirus transcription [21].

**Figure 1:**
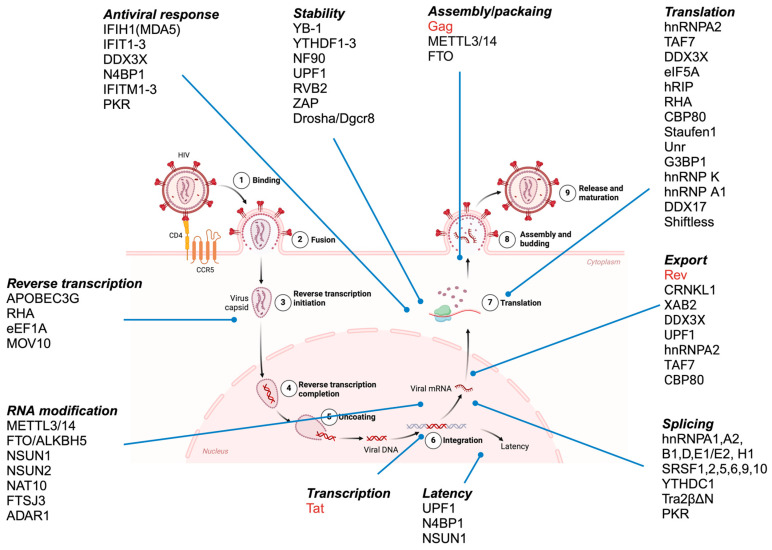
Overview of the HIV-1 replication cycle and the involvement of viral (red) and host (black) RNA-binding proteins in each step as described by targeted studies. After viral entry, viral RNA is converted into a proviral DNA integrated into the host genome. RNA polymerase ll mediates proviral DNA transcription to produce a 9 kb transcript, which can be fully spliced, partially spliced, or unspliced to produce viral proteins or packaged as genomic RNA (in the case of the unspliced RNA). RBPs are involved in every step of the viral cycle, including reverse transcription, transcription or latency, nuclear export or retention, translation, RNA modification, and packaging. Created with BioRender.com.

For Tat production, the basal transcription of the provirus must occur, producing the MS viral transcript. This RNA is expected to be exported from the nucleus by the nuclear export factor NFX1 in a canonical pathway similar to that of the cellular mRNAs, assembling RNP complexes like host mRNA. However, once Tat-mediated transcription begins, the intron-containing viral transcripts are exported by the viral protein Rev (also produced by the MS RNA), which binds to the Rev response element (RRE) in these transcripts and promotes their export by the CRM1 pathway. In this pathway, the RNPs in which viral transcripts are found differ in their composition from the RNPs formed by the MS RNAs. This includes the presence of RNA helicases such as DDX3X and UPF1 and the absence of splicing-dependent RBPs [22,23]. Finally, the NC domain of Gag recognizes the US RNA that is to be packaged as the viral genome, allowing the proper formation of the viral particle [24]. Although simplified here, the functions of these vRBPs are not limited to those mentioned above, as they are involved in a complex network of cell regulation through interaction with a plethora of host proteins [13]. In this section, we will describe examples of host RBPs that influence HIV-1 replication that have been identified through targeted studies (Figure 1).

### 3.1. Regulation of Viral RNA Splicing and Nuclear Export by Host RBPs

HIV-1 RNA splicing, like cellular transcripts, is a highly coordinated process, regulated by serine/arginine-rich (SR) proteins and heterogenous nuclear ribonucleoproteins (hnRNPs), which normally promote and inhibit splicing, respectively. RNA splicing involves the orchestrated activity of five small nuclear RNAs (snRNAs) and more than 100 proteins. It begins when the U1 snRNP binds to the 5′ splice site (5′ss) region in transcripts. Once bound, the spliceosome assembles, and through a complex process (reviewed in [25]), introns are removed, and exons are joined together. The HIV-1 full-length US transcript has several 5′ss regions, and the major donor site 1 (D1) located in the 5′ UTR can be used together with one of ten downstream acceptor sites to produce partially/singly spliced RNA (PS), or further splicing can take place at the D7-A7 sites to produce multiply spliced RNA (MS) [26]. Splicing is also regulated by the heterogeneous structure of the HIV-1 US transcript [27,28] and by the splicing regulatory elements (SREs) to which SR and hnRNPs proteins bind. A detailed description of the viral RNA splicing process of HIV-1 and the involvement of the hnRNP and SR proteins is reviewed in [29,30,31].

The viral RNA has several SREs, including exonic splicing enhancers (ESEs), intronic splicing enhancers (ISEs), exonic splicing silencers (ESSs), and intronic splicing silencers (ISSs) [9]. Approximately half of the transcripts during infection are US transcripts, and the remaining 50% are PS and MS viral RNAs, highlighting the finely regulated process of HIV-1 viral RNA processing. This is represented by mutations in the splice–donor (SD) hairpin, a stem-loop structure in the US RNA that can occlude the 11 nucleotides at which the U1 snRNA anneals that either stabilize or destabilize this structure, reducing HIV-1 expression and replication, indicating a highly regulated mechanism for viral expression [32,33].

In addition, SR protein binding sites present at the major 5′ splice site D1 of viral RNAs are of paramount importance for viral replication. Several SR proteins bind to these sites, particularly at the major donor site D1, to control viral RNA splicing, such as the enhancers SF2/ASF (SRSF1), SC35 (SRSF2), SRSF9, and SRSF10 [34,35] and the splicing silencers SRp40 (SRSF5) and SRp55 (SRSF6) [36,37], with the coordination of other cellular factors that interact with these SR proteins to carry out their function, such as DDX17 and the heterodimer U2AF65/35 [38]. Furthermore, the overexpression or silencing of these factors alters the ratio of the three types of viral transcripts, ultimately leading to a dramatic reduction in viral replication [39,40].

Heterogeneous nuclear ribonucleoprotein (hnRNP) siRNA screening in provirus-containing HeLa cells revealed that the depletion of hnRNPs A1 and A2 increased the viral expression of the structural proteins Gag and Env by affecting splice site selection and increasing the abundance of US viral RNA, respectively [41]. Furthermore, hnRNP D was found to be required for the efficient accumulation of US and PS viral RNA in the cytoplasm by only its p42 and p45 isoforms, without affecting the RNA decay rate, probably reflecting the involvement of this protein in the Rev-mediated export of US and PS viral RNA (vRNA) from the nucleus. hnRNP A2 has also been shown to play a role in HIV-1 RNA retention in the nucleus of HeLa cells through interaction with the hnRNP A2 response element (A2RE) located at the vpr coding region of the viral PS and US RNAs. When this region is mutated, the viral RNA is found predominantly in the cytoplasm. Furthermore, under hnRNP A2 knockdown, the vRNA that reaches the cytoplasm is found in translation-deficient RNP complexes. These data suggest that hnRNP A2 also plays a critical role in vRNA trafficking and translation, promoting the nuclear retention of vRNA and affecting the translation of the vRNA that reaches the cytoplasm [42].

Interestingly, the viral protein Tat co-opts hnRNPA2 in CD4^+^ T cells to achieve high levels of US RNA and its nuclear export. However, in astrocytes, Tat does not interact with this protein due to the low efficiency of Tat import into the nucleus. Therefore, there is an alteration in these RNA processes, which may explain why HIV-1 replication is highly restricted in astrocytes [43]. In the same study, the authors found that hnRNP A2 enhances vRNA splicing and the production of MS RNA in SupT1 (CD4^+^ cell line) and 1321N1 (astrocyte cell line) cells, similar to the finding of the siRNA screening mentioned above [41]. A further study assessed the binding of hnRNP A1, A2, B1, H1, and K to viral RNA by PAR-CLIP-seq in HEK293T cells and showed that hnRNP A1, A2, and B1 bind with low specificity across the HIV-1 US transcript, H1 binds only to discrete sites, and hnRNP K does not bind to the viral RNA, and confirmed that the depletion of any of these protein disrupts RNA splicing and alters viral replication [44]. Finally, hnRNP E1/E2 also binds to viral RNA at an ESS in the Tat/Rev exon 3, where it modulates the expression of the Rev viral protein, affecting overall viral MS RNA, protein expression, and replication fitness [45]. Tra2α/β and the splice variant Tra2βΔN are also cellular RBPs involved in the modulation of HIV-1 RNA splicing through interaction with ESE regions [46,47]. Tra2α/β overexpression leads to HIV-1 US accumulation, whereas the Tra2βΔN isoform leads to MS RNA accumulation, suggesting a contrasting role of splice variants of this protein in HIV-1 RNA processing and their potential use as target modulators of viral replication. This has been demonstrated by using the drug digoxin, which modifies Tra2β and reduces HIV-1 replication [48]. SRSF1, a splicing factor, has also been demonstrated to affect HIV-1 replication by binding to the TAR RNA motif, thereby excluding the Tat protein from the RNA. Furthermore, SRSF1 is also involved in splicing regulation, as the overexpression of the wild-type isoform causes an aberrant accumulation of US RNA and a drastic reduction in PS and MS RNAs, resulting in a 200-fold reduction in infectivity [49,50].

Interestingly, protein kinase RNA-activated (PKR) has also been reported to be involved in HIV-1 RNA splicing [51]. The activation of PKR modulates HIV-1 translation by the phosphorylation of eIF2α, leading to a partial shutdown in protein synthesis. During HIV-1 infection, the highly structured TAR region and a compact RNA pseudoknot upstream of the 3′ end of TAR can potentially activate PKR. Indeed, PKR is transiently activated in PBMCs during viral infection [52,53]. However, a co-immunoprecipitation analysis in HEK293T and confirmation in Jurkat cells revealed that PKR interacts with ADAR1 and the PKR activator (PACT). PACT interacts with the TAR-binding protein (TRBP), preventing PACT from activating PKR. Additionally, ADAR1 interaction with PACT leads to PKR inhibition through an unknown mechanism [52,53]. Furthermore, a recent study showed that the efficient splicing of Rev/Tat mRNA is dependent on specific eIF2α phosphorylation by PKR, which is activated by the recognition of viral transcripts (the TAR and pseudoknot structures) [51], similar to the enhancement of the RNA splicing of the transcript encoding the TNF-α protein, which also contains RNA structures that activate PKR [54]. Whether low-level activation of PKR persists during HIV-1 infection in the presence of PACT and ADAR1 and whether these low levels are sufficient to promote the efficient splicing of HIV-1 RNAs without affecting global protein synthesis remain to be elucidated.

On the other hand, the nuclear retention of vRNA in the nucleus is a complex process involving several cellular and viral factors. In this context, CRISPR/Cas9 screening has shown that CRNKL1 is an intron-containing vRNA retention factor through the direct binding of the vRNA, as determined by CRNKL1 immunoprecipitation analysis, without affecting splicing. XAB2 is another host factor that has been described in RNA splicing or the nuclear retention of the vRNA. However, its specific mechanism of action has not been described [55]. TAF7 is a nuclear factor that binds to cellular RNAs in the nucleus and contributes to their export to the cytoplasm and their delivery to polysomes. This protein shares several features with the Tat protein: both proteins have an arginine/lysine-rich basic region that recognizes RNA, recognizes similar adjacent elements (RNA delimited by a pyrimidine bulge and a steam loop), and also regulates transcription by interacting with TFIIH and *p*-TEFb to regulate the phosphorylation of the Pol II CTD. TAF7, like Tat, also binds to the TAR region in HIV-1 transcripts and assists in their export and translation [56]. However, the extent of the similarities and the role of TAF7 during HIV-1 replication remain to be fully elucidated.

Finally, RNA modifications, which involve the covalent attachment of chemical groups to nucleotides, are also involved in RNA metabolism, including the splicing process. The most common post-transcriptional or epitranscriptomic modification in eukaryotic mRNA and viral RNA is N6-methyladeosine (m^6^A), which can affect different steps of RNA metabolism depending on the reader protein recognizing the modification [57]. Antibody-dependent strategies coupled with next-generation sequencing including MeRIP-Seq [58,59] and PA-m^6^A-seq [60] revealed the presence of m^6^A along HIV-1 US RNA [58,59,60]. Although not all m^6^A sites are consistent between studies, the 5′UTR and especially the 3′UTR appear consistently modified in these studies. The nuclear m^6^A reader YTHDC1 was shown to negatively regulate HIV-1 alternative RNA splicing when it binds to the newly synthesized viral RNA, as its knockdown increases HIV-1 expression, especially for those transcripts with a D1/A1 splice junction, without affecting nuclear export or stabilization [61].

### 3.2. Cap-Dependent and IRES-Driven Ribosome Recruitment onto HIV-1 Transcripts Involves Host RBPs

Ribosome recruitment onto HIV-1 transcripts can occur via cap-dependent or IRES-driven mechanisms, depending on the physiological condition of the cell and the stage of the infection. As transcripts produced by RNA polymerase ll (Pol ll), HIV-1 viral transcripts harbor a 5′ cap structure and a 3′ poly(A) tail and use cellular translation machinery, like host mRNAs. However, unlike most host cell mRNAs, HIV-1 transcripts contain highly structured elements in their 5′UTR, particularly the TAR region, which is expected to interfere with ribosomal scanning and therefore require the assistance of additional proteins for cap-dependent translation initiation. Canonical translation factors that bind both mRNAs and HIV-1 viral transcripts will not be described here, but they have been reviewed elsewhere [62,63].

DEAD-box RNA helicase DDX3X is dispensable for general mRNA translation and interacts with HIV-1 RNA through RNA-specific determinants and the disordered regions of the protein [64,65]. When an in vitro-produced full-length HIV-1 RNA containing the cap structure transfected into HEK293T cells to bypass Rev-mediated export was used, it was shown that DDX3X, eIF5A, and hRIP are important cofactors required for US RNA translation in both cap-dependent and IRES-dependent translation [66]. Mechanistically, DDX3X binds to the 5′ UTR and unfolds the secondary structures (in this case, the TAR region) near the cap structure in an ATP-binding and hydrolysis-dependent manner, allowing the recruitment of the 43 S preinitiation complex and the initiation of the translation of HIV-1 transcripts [67] in an eIF4E- and CBP20/80-independent manner [68]. When DDX3X is depleted, HIV-1 translation is dramatically reduced while general translation is unaffected, demonstrating the specific role of DDX3X in viral translation. The DDX3X binding and unwinding of the TAR region appear to be mediated by the Tat viral protein, as both proteins interact and bind to TAR, thus facilitating viral RNA translation [69]. Another helicase important for the cap-dependent translation of HIV-1 transcripts is RHA (DHX9), which binds to the post-transcriptional element (PCE) in HIV-1 transcripts and induces their translation, as previously demonstrated by RHA silencing, which reduces de novo Gag synthesis without altering β-actin translation. This phenomenon was shown to be dependent on the helicase activity of RHA [70].

During transcription, nascent RNAs are capped at their 5′ end, which promotes interaction with the cap-binding complex (CBC). The CBC is required for efficient RNA processing, export, and the pioneer round of translation. It consists of CBP20, which binds to the cap, and CBP80, which stabilizes the cap–CBP20 interaction [71]. The RNA co-immunoprecipitation of CEM cells (a CD4^+^ T-cell model) showed that US and PS viral RNAs are associated with CBP80, leading to the active translation of these transcripts under cell cycle arrest induced by the accessory protein Vpr, despite the global reduction in the translation of cellular mRNAs, replacing the role of translation initiation factor 4E (eIF4E) [72]. However, as IRES-dependent translation was not assessed in this study, and cell cycle arrest was shown to favor this alternative mechanism of translation initiation [73,74,75,76], the true contribution of CBP80 under eIF4E downregulation requires further investigation. Nevertheless, CBP80 has been demonstrated to bind Rev and the US RNA in the nucleus and cytoplasm, supporting a role for Rev and CBP80 in nuclear export and translation [77]. A further study also revealed that the CBP80/20-dependent translation initiation factor (CTIF), which promotes interaction between CBP80 and the 40 S ribosomal subunit, competes with Rev for CBP80 binding and antagonizes the Rev–CBP80 complex-mediated translation of US RNA [78].

On the other hand, the IRES-driven translation initiation mechanism also contributes to the translation of HIV-1 vRNAs. Proteins that bind to this region located at the 5′UTR and promote its activity are called IRES transactivation factors (ITAFs). Several host factors have been classified as ITAFs for the HIV-1 IRES. Staufen1 is a protein found in RNP complexes involved in various aspects of mRNA metabolism, such as localization, translation, and decay. This cellular factor has pleiotropic effects during HIV-1 infection. It binds to viral RNAs at their 5′UTR to promote their translation in a cap-dependent manner [79] or at their IRES in a cap-independent manner acting as an ITAF [80]. Moreover, nearly 2–5% of Staufen1 bind to the Gag protein at the plasma membrane in an RNA-dependent manner to induce Gag oligomerization and the viral packaging of US RNA [81]. Under stress conditions, Staufen1 rescues vRNA from stress granules by dissociating this biomolecular condensate [82,83]. Therefore, the pleiotropic effects of Staufen1 make it a promising target for antiviral therapies.

The Unr protein has also been found to act as an ITAF for the HIV-1 IRES in HeLa cells, and its activity is regulated by Gag (p55) but not by its nucleocapsid product (NC, p7). However, the Unr-mediated activation of IRES-driven translation is mediated by the NC domain of Gag and is RNA-dependent [84]. Other reported ITAFs for HIV-1 IRES activity include eIF5A [85], hnRNP K [86], and hnRNP A1, the latter being upregulated during HIV-1 infection and retained in the cytosol, further enhancing HIV-1 IRES activity [87]. The Elav-like protein HuR is a negative regulator of the HIV-1 IRES, but this protein exerts its function by an unknown mechanism, as its control of ribosome recruitment onto the HIV-1 IRES was reported to be independent of its ability to bind to RNA [88].

Programmed ribosomal frameshifting (PRF) is a mechanism by which HIV-1 expresses the polyprotein Gag-Pol from its US RNA. This complex and highly regulated process involves the ribosome slipping into an alternative open reading frame in the slippery RNA element called the frameshifting stimulating element (FSE), which contains the sequence UUUUUUA [89]. This mechanism allows the maintenance of a critical Gag/Gag-Pol ratio of 20:1 [90]. In this context, the protein Shiftless (SFL) is a recently described HIV-1 restriction factor stimulated by interferon (IFN) type-I, -II, and -III. SFL interferes with Gag-Pol polyprotein synthesis by recognizing the translating ribosome and the FSE within the US viral RNA, abrogating the −1 programmed ribosomal frameshifting (−1PRF) process, resulting in premature translation termination [91]. However, it appears that HIV-1 infectivity restricted by SFL is not limited to −1PRF abrogation, as a mutant protein lacking the Zinc finger domain abrogates ribosome loading, SFL oligomerization, and binding to FSE-containing mRNA, but still reduces the infectivity of produced virions by an unknown mechanism [92,93]. In addition to participating in the ZAP-mediated degradation of vRNA (described below), DDX17 is also involved in the frameshifting of US RNA by an unknown mechanism, as its depletion leads to a 40% reduction in frameshifting efficiency [94].

The stalling of mRNA translation by the induction of stress granules (SGs) is a key feature of the cellular response to viral infection. SGs are translationally silent ribonucleoprotein structures containing multiple mRNAs and proteins such as G3BP1, TIAR, Staufen1, and several translational factors [95]. During HIV-1 infection, G3BP1 binds to the viral mRNA in the cytoplasm, sequesters the transcripts in SGs, and prevents the synthesis of viral proteins. However, the CA domain of the Gag polyprotein dissociates SGs, releasing the restriction imposed by these structures [96]. Nevertheless, there is evidence that G3BP1, through its ability to bind to viral RNA, restricts viral replication in primary macrophages and CD4^+^ T cells without the formation of SGs [97].

In addition to the m^6^A RNA modification, 5-methylcytosine (m^5^C) has also been shown to be involved in HIV-1 replication. This modification has at least eight “writers” and one “reader”. Among the “writer” proteins, NSUN1 (also known as NOP2) has been revealed to compete with the Tat protein for binding to the HIV-1 TAR region. Once bound to the TAR region, NSUN1 catalyzes the addition of m^5^C, which inhibits transcription elongation, promoting latency in different cellular models [98]. In sharp contrast, NSUN2 post-transcriptionally catalyzes the m^5^C modification of HIV-1 RNA and promotes ribosomal recruitment and viral mRNA translation, as demonstrated by Courtney et al., in which NSUN2 deletion resulted in reduced viral protein expression and alternative splicing without affecting the levels of viral RNA in CEM cells (CD4^+^ T cells) [99]. However, the results of this study have been challenged recently, as one study failed to detect any m^5^C modification on HIV-1 RNA [100]; another study that did detect m^5^C peaks in HIV-1 RNA revealed a minimal overlap between the m^5^C peaks found in the two studies [101]. Furthermore, Zhang et al. demonstrated that NSUN2 is involved in the IFN response of cells and that its knockdown promotes IFN-I activation, thereby enhancing antiviral cellular response [102]. Mechanistically, NSUN2 depletion alters the host m^5^C methylome and non-coding RNA (ncRNA) expression, favoring ncRNA transcription by RNA pol III, which can be recognized by RIG-I, a cellular pattern recognition receptor that triggers IFN production [102]. Therefore, elucidation of the actual implications of m^5^C for HIV-1 viral RNA and overall viral replication requires further studies.

Adenosine deaminase acting on RNA 1 (ADAR1) is an RNA-editing enzyme with antiviral activity against several RNA viruses. However, in HIV-1 replication, this enzyme seems to be a proviral host factor, since its depletion in Jurkat cells or infection of ADAR1-deficient CD4^+^ T cells from patients with Aicardi–Goutières syndrome inhibits viral replication at the level of Gag mRNA translation, with the concomitant activation of interferon-stimulated genes [103]. ADAR1 binds to the 5′UTR of viral transcripts and edits adenosines in this region [104]. Although the ADAR1-mediated enhancement of viral replication is editing-dependent, it can also be editing-independent, probably through PKR inhibition [105]. However, some studies on HEK293T and Jurkat cells also concluded that ADAR1 is an antiviral factor by inducing A-to-G mutations in Rev RNA, the RRE region of viral transcripts, and in Env mRNA, thereby reducing viral replication [106]. Further studies are therefore needed to clarify whether and when ADAR1 is an antiviral or proviral host factor.

### 3.3. Host RBPs Involved in mRNA Stability and Decay Regulate the Fate of HIV-1 Transcripts

Cells have several quality control mechanisms to ensure that gene expression is carried out correctly. These mechanisms include the exclusion of intron-containing RNA from nuclear export, translation-induced nonsense-mediated mRNA decay (NMD), No-Go decay (NGD), non-stop decay (NSD), or degradation by the RISC complex via miRNAs. Regardless of how the RNA structure or sequence alteration is detected, degradation involves the removal of the 3′ poly(A) tail (deadenylation) and the cap structure (decapping) [107]. In the former, the protein PABPC1 bridges the poly(A) tail with the Pan2/3 complex, which removes the distal part of the tail. The CCR4-NOT complex then digests the remaining adenosines. With the tail removed, the exosome complex can bind to the mRNA and digest it in a 3′–5′ direction. For decapping, the DCP1/2 complex binds to and removes the cap structure, allowing the XRN1 or XRN2 in the cytoplasm or nucleus, respectively, to digest the mRNA in a 5′–3′ direction [107,108]. As RNA Pol-II transcripts with a poly(A) tail and a cap structure, HIV-1 transcripts are a substrate for host RNA decay machinery and therefore are also controlled at this level. HIV-1 interaction with this decay machinery has been reviewed in [109].

Up-frameshift protein 1 (UPF1) is a key factor in nonsense-mediated mRNA decay (NMD), a process that degrades RNAs with premature stop codons from cells. UPF1 interacts with UPF2, UPF3A, UPF3B, SMG1, and the endonuclease SMG6. UPF1 forms RNPs with HIV-1 RNA to promote its stability and nuclear export and to enhance Gag protein translation, a process that requires UPF1 ATPase activity and is UPF2- and NMD-independent [110,111]. However, it is not clear whether UPF1 interacts directly with viral RNA or indirectly through interaction with Rev (in the nucleus) and Gag (in the cytoplasm). A further study also demonstrated that both UPF1 and UPF2, as well as SMG6, are involved in HIV-1 provirus reactivation from latency, as UPF1 knockdown in J-Lat cells stimulated with PMA resulted in a reduction in almost 70% of viral RNA measured by RT-qPCR in nuclear and cytoplasmic fractions, suggesting a role in viral RNA stability rather than nuclear retention. Conversely, the overexpression of UPF1 led to enhanced HIV-1 reactivation by increasing viral RNA levels [112]. Another study showed that UPF2 and SMG6 reduce HIV-1 expression and replication in monocyte-derived macrophages (MDMs) by downregulating viral transcript levels. In contrast to results regarding CD4^+^ T cells, this study found no changes in viral transcript levels upon UPF1 depletion [113].

The DNA- and RNA-binding protein Y-box binding protein 1 (YB-1) has been described as a cellular cofactor in early and late events of viral replication. It enhances RNA stability by binding directly to the YB-1-responsive element located at stem-loop 2 in the HIV-1 RNA packaging signal (5′UTR), thereby increasing viral protein expression [114]. In addition, the knockdown of YB-1 restricts viral replication by interfering with an as-yet-unknown process in reverse transcription or nuclear import [115]. Nuclear factors 45 (NF45) and 90 (NF90) have been identified in proteomic studies as positive host factors for HIV-1 replication [116]. These proteins are RBPs that are involved in RNA metabolism. In the context of HIV-1 infection, NF90 binds to the 3′UTR of cyclin T1 mRNA and enhances its expression, which in turn promotes HIV-1 transcription and replication [117]. The overexpression of NF45 and NF90 increases HIV-1 replication in HEK293T and HeLa cells by directly binding to viral RNA without significantly increasing cyclin T1 expression. However, only NF90 was revealed to be involved in increasing the half-life of HIV-1 RNAs [118]. RuvB-like 2 (RVB2) has been described as a host cellular factor that fine-tunes the HIV-1 translation of US RNA during replication in HEK293 cells by specifically binding to the 5′UTR of this mRNA and to the matrix (MA) region of the nascent Gag polyprotein, leading to ribosome stalling and the eventual decay of the translating viral mRNA. Interestingly, the C-terminal domain of the Env protein (which is present in the cytosol) relieves the inhibition imposed by RVB2 and increases the stability of US RNA by competing with RVB2 for the MA in the nascent polyprotein. Therefore, RVB2 seems to be involved in the regulation of viral particle production and infectivity by maintaining a correct Gag/Env ratio [119].

Other cellular factors involved in viral RNA decay are the microprocessor complex (composed of Drosha and Dgcr8), the protein ZAP, and RNA modifications. The microprocessor complex is involved in the regulation of miRNA levels and was shown to be responsible for RNA Pol ll pausing and the premature termination of proviral transcription in the absence of Tat in HeLa cells with an integrated HIV-1 proviral DNA. This mechanism involves the recruitment of this complex to the TAR region, where Drosha cleaves TAR and recruits the proteins Setx, XRN2, and Rrp6, which further degrade the nascent viral transcript [120]. The decay machinery thus controls HIV-1 replication from the very first transcription steps, from proviral DNA to the expression of late mRNAs encoding the structural polyproteins Gag and Gag-Pol.

The Zinc finger antiviral protein (ZAP or ZC3HAV1) is a host factor that recognizes RNA sequences with a high proportion of CG dinucleotides, which are suppressed in the genomes of HIV-1 and many other vertebrate viruses [121]. Once bound to HIV-1 RNA, ZAP inhibits translation and targets the RNA for degradation, thereby limiting viral replication in a process regulated by the E3 ubiquitin ligase TRIM25 [122]. Mechanistically, ZAP recruits a protein containing a ribonuclease domain called KHNYN [123], which cleaves the bound RNA. Although HIV-1 suppresses CG dinucleotides, susceptibility to ZAP has been mapped to a 700-base-pair region in the 5′UTR of the Env mRNA in primary HIV-1 strains, resulting in reduced Env protein expression and reduced viral infectivity [124]. Another host factor involved in HIV-1 restriction is SAM domain- and HD domain-containing protein 1 (SAMHD1), which has been largely described as a potent HIV-1 restriction host factor that acts by depleting the intracellular pool of dNTPs, thereby dramatically reducing reverse transcription. However, this factor also binds to single-stranded RNA, leading to its oligomerization on the bound RNA molecule and stimulating its ribonuclease activity [125,126]. The depletion of SAMDH1 by siRNA in macrophages and CD4^+^ T cells from healthy donors increases HIV-1 RNA stability and overall viral replication. Furthermore, the RNase domain but not the dNTPase domain of this factor is essential for HIV-1 restriction [127].

Finally, RNA modifications also play a key role in viral RNA stability and decay. The cytoplasmatic m^6^A readers YTHD1-3 are able to bind to the 3′UTR to enhance HIV-1 RNA accumulation in 293T and CD4^+^ CEM-SS T cells [60]. Mechanistically, it was proposed that YTHD2 binds to the 3′UTR of viral RNAs and stabilizes them, which is in contrast to its functions promoting the decay of m^6^A-containing cellular mRNAs [61]. However, another study found that the overexpression of YTHD1-3 in HeLa/CD4, Jurkat cells, and primary T cells restricted HIV-1 replication in the early steps, whereas their KO increased HIV-1 expression [58,128]. Despite the conflicting results of these studies, it is clear that the m^6^A cytoplasmic reader proteins are key players during HIV-1 replication, and further studies are needed to elucidate their role and molecular mechanism during infection in different cell types. One proposed hypothesis is that YTHD1-3 have a bimodal and contrasting role during HIV-1 replication. As such, during the early steps of viral replication, these m^6^A reader proteins bind to the incoming US RNA, leading to its degradation and decreasing reverse transcription and hence viral replication [128]. However, during the late steps of replication, these same proteins recognize m^6^A in viral transcripts and target it for translation [58]. The m^6^A writers METTL3/METTL14 and the erasers FTO and ALKBH5 are also important players during HIV-1 infection, as these proteins control the m^6^A dynamics of cellular and viral transcripts. Indeed, the silencing of METTL3/14 and ALKBH5 decreased and increased viral replication, respectively. However, m6A-mediated viral expression is not limited to YTHDF1-3 proteins, as the presence of two actively methylated adenines (A7877 and A7883 of the HIV-1 LAI strain) has been demonstrated at the IIB region of the RRE hairpin that promotes Rev–RRE complex formation during infection and thus RRE-containing viral RNAs nuclear export [59]. The effects of m^6^A on HIV-1 replication have recently been reviewed [129].

N4-acetylcytidine (ac4C) is a lesser-known epitranscriptomic mark deposited by the N-acetyltransferase 10 (NAT10) enzyme. It involves the covalent attachment of an acetyl group to the cytidine residues instead of methylation events. Through the use of SupT1 and CEM infected cells (both models of CD4^+^ T cells), it was demonstrated that the HIV-1 RNA has this modification in its CDS and 3′UTR and that NAT-10 catalyzes this process [130]. In this context, it was shown that ac4C enhances Gag expression by increasing the stability of US viral RNA without affecting its translation. Interestingly, in some interactome studies, NAT10 has been found to be an interaction partner of the viral regulatory proteins Rev [12,131] and Tat [131]. These proteins probably mediate the recruitment of the acetyltransferase to the HIV-1 US RNA, as NAT10 has also been identified as an HIV-1 RNA interactor [132]. Another RNA modification is 2′-O-methylation (2′-O-Me), which is a marker that allows the cell to distinguish its own RNAs. The TAR RNA-binding protein (TRBP) binds to FTSJ3, the 2′-O-methyltransferase (2′O-MTase) responsible for adding this modification to cellular RNA, and promotes the interaction of FTSJ3 with the viral transcripts. This modification allows HIV-1 RNA to evade MDA-5 and RIG-I sensing and IFN-I production [133] but also allows the evasion of antiviral mechanisms such as viral RNA degradation by the RNAse ISG20 [134].

### 3.4. Packaging and Early Infection Steps

As mentioned above, m^6^A deposition by METTL3/14 leads to the increased stability of the viral RNA, which also leads to the increased production of the Gag protein. However, it has been demonstrated in HEK293T cells that two populations of US RNA coexist: one methylated at the 5′UTR (of the NL4.3 molecular clone), which promotes its translation, and the other with these adenosines unmodified, promoting its packaging and therefore its use as genomic RNA. The coexistence of these two populations is likely maintained by a balance between m^6^A deposition by METTL3/14 and m^6^A removal by the eraser FTO [135]. However, the specific mechanism by which the lack of m^6^A at the 5′UTR leads to the US RNA being packaged and whether the YTHDF1-3 readers are involved in these processes remain elusive.

Cytidine deaminase members of the apolipoprotein B mRNA-editing enzyme-catalytic polypeptide-like 3 such as APOBEC3G (A3G) are packaged within viral particles. Once US RNA has been converted into DNA, this enzyme causes hypermutations in the newly produced viral DNA by converting cytidines to uridines, leading to G-to-A mutations and dramatically reducing HIV-1 infection in the next infectious cycle [136]. However, the accessory protein Vif binds to A3G and promotes its degradation. Although A3G binds to viral DNA, recent in silico studies have demonstrated that A3G binding to the US RNA used as genomic RNA is critical for the packaging of this protein but also in recognition by Vif. Accordingly, it is hypothesized that Vif does not recognize A3G alone but recognizes the A3G–US viral RNA complex [137]. A3G–US RNA interaction has also been associated with reduced viral DNA synthesis by an editing-independent mechanism through the steric inhibition of viral reverse transcriptase, altering DNA elongation [138,139]. However, a further study showed that direct interaction between A3G and HIV-1 reverse transcriptase is required to inhibit viral DNA elongation and that A3G binding to US RNA is dispensable [140].

The primer binding site (PBS) in the 5‘UTR of viral genomic RNA is a key region where reverse transcription begins. This region folds into a three-way junction structure consisting of a tRNA-like element (TLE), the tRNA annealing arm, and the primer activation signal (PAS) [141]. Proper folding is critical for the loading of host factors required for reverse transcription, such as DHX9/RNA helicase A (RHA). RHA is critical for the unwinding of secondary and tertiary structures in genomic RNA, therefore promoting the elongation phase of reverse transcription. The removal of RHA or the loss of the folding structure of the PBS dramatically reduces HIV-1 infection [141,142].

eEF1A is packaged into viral particles, where it binds to the 5′ UTR of US RNA, helping in the late reverse transcription stage of DNA synthesis. Its binding domain is mapped to nucleotides 142 to 170 [143]. Accordingly, eEF1A depletion in HEK293T leads to reduced viral replication through a reduction in HIV-1 DNA synthesis [144]. Interestingly, this site overlaps with the human lysyl-tRNA synthetase (hLysRS) binding site [145], a process required for the release of the primer tRNA, allowing the reverse transcription process to begin. As both proteins are incorporated into viral particles and both recognize tRNA [143,145], an answer to the question of how these proteins compete or interact needs further investigation.

### 3.5. Host RBPs in the Context of the Antiviral Response

Infected cells produce pro-inflammatory cytokines during the infection of dendritic cells, macrophages, and CD4^+^ T cells. However, the cellular sensor of the virus is largely unknown [146,147]. In macrophages, interferon-stimulated gene (ISG) induction has been described to occur through two events. The first occurs 1–4 h after infection and is associated with extracellular vesicles produced by producer cells and controlled by IRF1. The second induction occurs 3 days after infection and is triggered by viral mRNA via the activation of the RIG-I/MAVS axis without IFN-I production. This later response is commanded by IRF7, which is itself an ISG produced by IRF1 during the earlier response [148]. In this study, the single knockdown of IFIT1-3 was shown to enhance HIV-1 replication in monocyte-derived macrophages (MDMs) [148]. Similarly, another study using MDMs and monocyte-derived dendritic cells (MDDCs) found that the post-integration steps of HIV-1 infection trigger IFN-β mRNA production 3 days post-infection, with a concomitant increase in the ISGs CD169 (also known as Siglec1) and IP-10 (CXCL10), but a low level of IFN-β production with a negligible effect on HIV-1 spread was reported. Furthermore, CD169 induction was found to be dependent on Gag association with the plasma membrane, with the possible involvement of NF-κB, TAK1, and mitochondrial antiviral-signaling proteins (MAVSs) but not STING, MDA5 (IFIH1), or RIG-I (DDX58) in this activation [149]. However, the specific knockdown of twenty-one candidate genes as possible HIV-1 RNA sensors revealed that CRM1 (XPO1), MDA5, and MAVS abolished IFN-I pathway activation in MDMs and MDDCs, as determined by an intracellular flow cytometry evaluation of ISG15. The formaldehyde cross-linking immunoprecipitation (fCLIP) of MDA5 revealed the enrichment of US but not PS or MS viral RNAs [150]. These data suggest that intron-containing US RNA is what myeloid cells sense and respond to during HIV-1 replication, but further studies are needed to fully elucidate the sensor(s) protein(s) during viral replication.

In this context, DDX3X has also been proposed as such a sensor in dendritic cells. During HIV-1 replication, several abortive transcripts are generated as errors during proviral DNA transcription. These transcripts are detected in dendritic cells by DDX3X, which induces IFN-I production via MAVS. However, the DC-SIGN sensing of HIV-1 inhibits TRAF3 binding to MAVS, thereby attenuating the DDX3X-MAVS pathway [151]. In turn, DC-SIGN signaling enhances proviral DNA transcription by inducing phosphorylation at Ser276 of the p65 subunit of the NF-κB heterodimer, which is activated by toll-like receptor 8 (TLR8) signaling in dendritic cells as a response to viral single-stranded RNA [152]. Accordingly, TLR8 activation in CD4^+^ T cells not only activates these cells and enhances viral replication by boosting T-cell receptor (TCR) signaling but also potentially reactivates the virus from latently infected cells [153]. Although several DEAD-box helicases (DDXs) are involved in different aspects of viral replication, they exert their function in the context of HIV-1 infection through protein–protein interaction, with their ability as RBPs often being dispensable. These DDXs include DDX1 [154], DDX5 [155], DDX6 [156], and DDX21 [157]. Comprehensive reviews of DDX helicases during HIV-1 replication can be found in [158,159]. On the other hand, HIV-1 appears to regulate TLR3 signaling by inhibiting the phosphorylation of IRF3/IRF7, STAT1, and STAT2 [160] and by downregulating IRF3 protein levels [161]. There are also reports showing that HIV-1 RNA is recognized by plasmacytoid dendritic cells via TLR7, activating them and inducing the maturation of bystander myeloid cells [162,163,164]. However, this phenomenon appears to be specific to this cell population.

The screening of 62 antiviral sensor RBPs in HEK293T cells by the co-transfection of the corresponding RBP-encoding plasmids and the pNL4.3 plasmid revealed a 20-fold reduction in the infection of TZM-bl reporter cells by viral particles from cells transfected with the NEDD4-binding protein 1 (N4BP1) construct. N4BP1 is an ISG that binds to all three viral transcripts in Jurkat cells and primary macrophages. Interestingly, this HIV-1 restriction factor is not counteracted by any of the viral accessory proteins but by MALT10, a long non-coding RNA (lncRNA) induced after T-cell activation, which degrades N4BP1 and leads to the reactivation of the virus in latently infected J-Lat cells [93]. No other sensor proteins were found in this study.

Interferon-induced transmembrane proteins (IFITMs) are well-documented antiviral factors. Three types of these proteins are encoded in humans: IFITM1, IFITM2, and IFITM3. The mechanism of action of these antiviral factors is based on the inhibition of viral entry and reduction in viral infectivity by co-packaging within virions, as IFITM proteins interact with and alter the physical properties of the plasma membrane, inhibiting virus–cell membrane fusion [165]. In particular, CCR5 strains are more sensitive to IFITM1, whereas CXCR4 strains are more susceptible to IFITM2 and IFITM3 inhibition [166,167]. However, inhibition by IFITMs has been shown to not only limit HIV-1 viral entry but also exclude viral RNA from polysomes, thereby reducing HIV-1 protein expression. The transfection of a codon-optimized vector for HIV-1 NL4.3 Gag, which changes the codon bias to humans and alters viral RNA structure, is not readily recognized by these proteins, and viral inhibition is not only recovered but enhanced. Interestingly, it was demonstrated that the viral protein Nef helps to overcome the IFITM1 restriction imposed on HIV-1 RNA translation [167]. However, it was not determined why only US and PS RNAs were affected, as this effect was RRE-independent.

In summary, several cellular proteins recognize HIV-1 RNA and trigger the production of a limited number of cytokines. However, the majority of these proteins are counteracted by viral or cellular molecules. Interestingly, although ISGs can be detected in some cellular models upon HIV-1 infection, interferons are usually not produced, and the induced ISGs are not sufficient to eradicate the virus.

## 4. Unbiased Proteomic Studies of HIV-1 RBPome

While individual, targeted genetic and proteomic studies have significantly advanced our understanding of the various roles that cellular RBPs play in HIV-1 biology, unbiased proteome-wide studies have emerged in recent years as a critical approach to mapping the RBPome across different viruses and cell types, including SINV [168,169], ZIKV [18,170], CHIKV [171], DENV [18,172], SARS-CoV2 [20,173,174,175], and HIV-1 (discussed below). These studies have employed RNA affinity capture strategies in both in vitro-transcribed HIV-1 RNA segments (such as the 5′UTR) and the entire RNA captured during infection. In the former, cell lysates are incubated with HIV-1 RNA segments, or cells harboring a modified proviral DNA are stimulated for transcription and cells are then lysed. In both cases, the viral RNAs contain a tag used for RNA purification. The RBP-bound RNAs are captured using specific methods (described below) and purified using magnetic beads, followed by washing, elution, and analysis by mass spectrometry.

**Figure 2:**
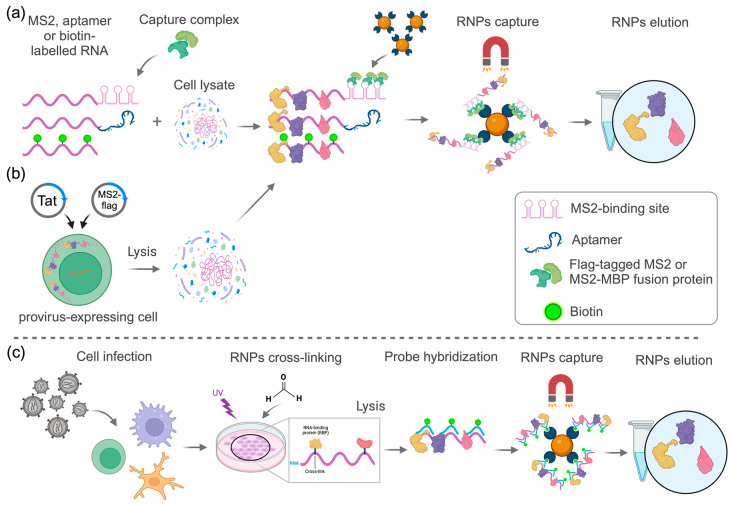
Approaches used to study the HIV-1 RBPome in different models. In vitro-transcribed fragments of HIV-1 RNA tagged with biotin, MS2-binding sites, or an aptamer are incubated with a cell lysate (**a**); or U2OS cells containing a provirus encoding truncated fragments of HIV-1 RNA tagged with MS2-binding sites are transfected with a Tat-encoding plasmid to induce provirus reactivation and with an MS2-encoding plasmid to produce flag-tagged MS2, and cells are lysed (**b**). In both cases, the viral RNAs bound to the RBPs are captured and purified using magnetic beads coupled to streptavidin (for biotin-tagged transcripts) or an anti-flag antibody (for flag–MS2 bound transcripts), or captured by an MS2–maltose-binding-protein (MBP) fusion protein or a tobramycin-coupled agarose matrix. After capture/purification, samples are eluted and analyzed by mass spectrometry. Alternatively, cells are infected for a pre-determined time prior to RNA–protein cross-linking by UV light or formaldehyde, and cross-linked RNPs are hybridized with specific HIV-1 RNA biotin-labeled antisense probes and captured/purified with streptavidin-coupled magnetic beads or hybridized and captured with oligo(dT)-coupled magnetic beads. Captured RNPs are eluted and analyzed by mass spectrometry (**c**). Created with BioRender.com.

In the second RNA affinity capture approach, cells are infected with wild-type, single-round, pseudotyped, or other replication-competent viruses, and RNA–protein interactions are cross-linked or “frozen” either by exposure to UV at 254 nm or 365 nm (with photoactivatable, modified ribonucleotides) to capture protein interactions at “zero” distance or with formaldehyde, which preserves both direct and indirect RNA–protein interactions. After cross-linking, cells are lysed, and vRNA is isolated by hybridization to labeled antisense probes (typically biotin-labeled) or poly(dT) probes. The hybridized RNA is then captured using magnetic beads, followed by washing, elution, and analysis by mass spectrometry (Figure 2). These methods have revealed an extensive network of RNA-interacting proteins, providing deeper insights into the intricate interactions between viruses and host cellular factors. The key advantages of these approaches include the preservation of natural, physiological interactions between viral RNA and its interacting proteins, the use of relevant cell types (i.e., those naturally targeted by the virus), and the high specificity of detected interactions due to the cross-linking step and the rigorous, highly denaturing washing conditions.

### 4.1. HIV-1 RBP Assessment Based on Interactomes of Viral RNA Segments

This approach has been used by Kula et al. to investigate the proteins that interact with HIV-1-derived vectors. These vectors consisted of truncated viral RNAs modified with 24 MS2-bacteriophage-binding sites. The authors transfected U2OS carrying the stable vectors with a Tat plasmid (Tat-CFP) to induce proviral transcription and a flag-tagged-MS2-coating-protein-coding plasmid (Flag-MS2nls), cells were lysed, and the complex RNA–MS2 coating protein was purified using anti-flag-coupled beads and analyzed by mass spectrometry [176]. This approach revealed 32 RNA interactors, several of which were previously known to interact with HIV-1 RNA, such as splicing factors, NF90, DBPA, RPL3, DDX3X, SFPQ, PTB, and UFP1. Other interesting proteins found were MOV10, an RNA helicase with antiviral activity, and GAPDH, and the involvement of MATR3 in HIV-1 RNA metabolism was discovered. Mechanistically, MATR3 was found to be in a complex with Rev and RRE-containing RNA, and its depletion resulted in a marked reduction in cytoplasmic levels of unspliced RNA, suggesting that this protein is a key player in Rev-mediated unspliced RNA export from the nucleus [176,177]. Furthermore, MATR3 is also a positive post-transcriptional regulator of HIV-1 acting on US RNA translation [178], and a negative inhibitor of ZAP through the recruitment of the ZAP degradation complex (DDX17 and EXOSC3) in an RNA-dependent manner, demonstrated by the depletion of MATR3 leading to ZAP restricting HIV-1 replication through the recognition of not only multiply spliced RNA (its target when MATR3 is present) but also the other viral transcripts [179].

In order to identify new protein partners involved in HIV-1 RNA splicing by using the A7 splice site, Marchand et al. incubated HeLa cell nuclear extracts with the HIV-1 Tat/Rev exon 3 5′-terminal region, which folds into a stem-loop called SLS2-A7, bound to three MS2-binding sites (SLS2-A7-MS2) or an aptamer with high affinity for tobramycin (SLS2-A7-J6f1) [180]. The formed RNPs were purified by the MS2–maltose-binding-protein (MBP) fusion protein or by tobramycin-coupled agarose matrix, respectively, followed by mass spectrometry analysis. The MS2 and j6f1 tag purification yielded 33 and 24 protein partners, respectively. These partners included several splicing regulatory proteins such as HNRNP A1, A3, and H, as well as other proteins such as nucleolin, NF45/90, and several DNA-binding proteins. Interestingly, this study identified HNRNP K as a new important HIV-1 splicing regulator [180].

RBPs have also been implicated in HIV-1 latency control. An unbiased proteomic study revealed 243 proteins interacting with Tat mRNA, 132 in TNF-stimulated and 96 in unstimulated JLAT6.3 cells (an HIV-1 latency model based on Jurkat cells). When some of these proteins were validated by a dual-fluorescent reporter virus (DuoFluo, R7GEmTB) infection in knockdown Jurkat cells, all thirteen proteins were involved in either latent (TOP2A, SRP14, HNRNPH1, DDX1, and HNRNPL) or productive infection (FLNA, HMGB3, PTBP1, HSP90AA1, and KIF2C), with SRP1 knockdown decreasing splicing while HMGB3 and PTBP1 increased it. In addition, footprinting assays coupled with SHAPE revealed that SRP1 and HMGB3 bind to the SLS3A3 region of Tat mRNA, which includes the start codon and the TIM-TAM region, suggesting that SRP1 and HMGB3 are regulators of Tat production and therefore of the latent and productive cycles, respectively. Interestingly, the overexpression of SRP1 in J-Lat cells and CD4^+^ T cells purified from PLWH reactivates the provirus without activating the cell, highlighting the potential role of Tat mRNA-binding proteins in latency reversal [181].

**Figure 3:**
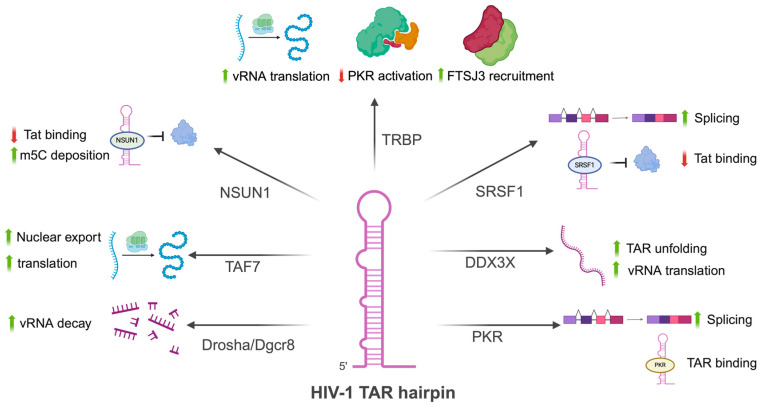
The HIV-1 transactivation response (TAR) element is a hotspot for host RNA-binding proteins. Several host proteins bind to the HIV-1 5′UTR, many of which mediate important functions by binding to the TAR region. These proteins include TAR RNA-binding protein (TRBP), which enhances viral mRNA translation, inhibits PKR activation, and recruits FTSJ3, which deposits the epitranscriptomic mark 2′-O-methylation, allowing the vRNA to be recognized by the cell as a self-molecule. NSUN1 binds to the TAR region, blocking Tat binding, which reduces HIV-1 transcription and promotes latency, and deposits the m^5^C mark, likely leading to transcript degradation. TAF7, a host protein similar to Tat, promotes the nuclear export and translation of vRNA. The microprocessor complex Drosha/Dgcr8, when bound to TAR, leads to the recruitment of RNA decay machinery and vRNA degradation. SRSF1 also reduces Tat binding and promotes US RNA splicing. Once the vRNA reaches the cytoplasm, DDX3X unfolds the highly structured TAR region, allowing vRNA translation. Finally, PKR binds to the TAR region, where it could lead to global translation arrest, but is counteracted by ADAR1, TRBP, and PACT. Instead, low-level PKR binding to the TAR region likely leads to vRNA splicing enhancement through eIF2α phosphorylation. Created with BioRender.com.

A previous study identified 185 RBPs associated with the 5′UTR of HIV-1, spleen necrosis virus, and Rous sarcoma virus, out of which 122 were previously reported to be involved in retroviral replication and 63 were newly identified RBPs interacting with the retroviral 5′UTR. Among these proteins, 122 corresponded to HIV-1 5′ UTR interactors, with a predominant role in transcription/mRNA processing/splicing, followed by translation factors and rRNA biogenesis and processing factors. Several of the identified proteins corresponded to double-stranded RNA-binding domain (dsRBD)-containing factors such as DHX9, DHX30, DDX3X, DDX17, DDX5, Staufen1, PKR, and NF90. Furthermore, proteins such as MATR3, the splicing factor SFPQ (PSF), NONO, and nucleolin (NCL) were also identified. As these proteins bind to the 5′UTR, several of them could be ITAFs, but IRES activity was not evaluated [182]. The results from this study suggest that the 5′ UTR of HIV-1 represents a hotspot for viral RNA metabolism control by cellular RNA-binding proteins, several of which are likely to be mediated by the TAR region (Figure 3).

### 4.2. RNA Affinity Capture Studies for HIV-1 RBP Assessment During Infection

Knoener et al. developed a new method to interrogate the HIV-1 US transcript interactome by designing a biotinylated DNA oligonucleotide capture probe and a release probe [183]. Briefly, Jurkat cells were infected with VSV-G-pseudotyped HIV-1 (NL4.3 with disruptive mutations in the Env, Vpr, and Nef genes), cross-linked with formaldehyde, and lysed. RNPs were hybridized by incubation with the capture probes and captured with streptavidin-coupled magnetic beads. Beads were washed under denaturing conditions, and RNPs were assessed by incubation with the release probe, which is more thermodynamically favored for hybridization with the capture probe, releasing the target RNA. A proteomic analysis revealed 189 US RNA interactors, the majority of which were RNA metabolism-related proteins, including splicing, transcription, nucleocytoplasmic transport, and translation factors [183].

This work was further developed by individually assessing the RNA interactome for each of the three classes of viral RNA transcripts—US, PS, and CS—by the incorporation of a probe against intron-1 (for US), intron-2 (for PS), or the 3′ exon (present in all transcripts) in a sequential manner from the same Jurkat cell total lysate [132]. Over 900 common protein interactors were found for all three viral transcripts. Of these interactors, nearly 630 proteins have been previously identified as interacting with polyadenylated cellular mRNA, suggesting that, as expected for RNA polymerase II-transcribed RNAs, the majority of RBPs interacting with HIV-1 transcripts serve general roles in mRNA metabolism such as in splicing, export/transport, stability, localization, and translation [184,185]. Importantly, this study found the majority (but not all) of the proteins found in the previous HIV-1 RNA interactome evaluation, thus validating their results. Furthermore, 102 proteins were previously unknown interactors of HIV-1 transcripts, including five proteins not previously implicated in RNA regulation: TRIM56, BUB3 (mitotic checkpoint protein), Dynamin 2 (DNM2), Dynein cytoplasmic 1 heavy chain 1 (DYNC1H1), and Nicalin (NCLN), highlighting the great value of RNA interactome evaluation. An siRNA evaluation of 121 identified proteins revealed an alteration in HIV-1 replication for 84 proteins (69%), validating the importance of these proteins in viral replication. Interestingly, 212 proteins were found to differentially interact with at least one transcript class: 101, 93, and 68 interactors for US, PS, and CS, respectively. Notably, the US RNA-specific interactors included proteins known to be mitochondrial proteins (8 proteins) or known to be involved in carboxylic acid metabolism (19 proteins). Further studies are needed to characterize RNA-binding proteins with metabolic functions that bind to HIV-1 viral RNA.

RBPs have also been studied in the context of their incorporation into viral particles. Garcia-Moreno et al., 2023 used the in-virion RNA interactome capture (ivRIC) approach, in which the RNA and the proteins of purified virions are cross-linked with UV light and captured with an oligo(dT) probe, pulled down, and analyzed by mass spectrometry [186]. This approach revealed that 104 host proteins were not randomly incorporated into the HIV-1 virions produced by SupT1 cells (a model of CD4^+^ T cells), and, interestingly, many of these were proteins with nuclear localization. In all the above studies, HIV-1 gene expression, infectivity, or RNA stability is altered when some (but not all) of the identified RBPs are depleted, such as MOV10, HNRNPC, and HSPD1 in HEK293T cells and MSN, FAM120A, PURA, and PURB in SupT1 cells. This study raises the question of whether HIV-1 viral particle composition varies among different producer cells, and whether these differences have implications in the experimental setting or in natural infection when studying HIV-1 replication, as different proteins can be packaged in the virion and this differential composition can influence the success of the new infection cycle in different manners.

Taken together, these data indicate that (i) an important number of cellular proteins interact directly with HIV-1 US RNA (RNA-binding proteins); (ii) host proteins interact with the US RNA inside cells, and some of these proteins remain associated with the viral US RNA and are incorporated into virions that carry the proteins to regulate the next infection cycle; (iii) understanding the function of viral proteins is not sufficient to fully understand the molecular pathways that HIV-1 hijacks during infection; and (iv) the implications of RBP-HIV-1 RNA interactions are far more complex than previously thought, and their study will potentially provide new therapeutic targets to treat HIV-1 infection.

## 5. Concluding Remarks

RNA-binding proteins (RBPs) are active players during the HIV-1 replication cycle, from its entry into cells to the production of viral particles, affecting processes such as the transcription of the provirus, nuclear retention or export, stability or decay, translation, and the modulation of cellular pathways. Our knowledge of the HIV-1 RBPome and its role in viral replication has been greatly expanded by the introduction of unbiased approaches allowing the identification and characterization of these proteins. Nevertheless, the HIV-1 RBPome has not been fully elucidated, nor have the implications of all the described RBPs for the viral replication of this virus. There is a lack of RNA interactome studies on different natural targets of HIV-1, such as subtypes of T cells or myeloid cells such as monocytes, tissue macrophages, or microglia, and in different cellular contexts such as the RBPome during HIV-1 provirus reactivation from latency. Furthermore, the refinement and validation of the currently available strategies for RBPome determination are needed to implement these protocols in the study of the RBPome of tissues from people living with HIV, as has recently been developed for the RNA interactome evaluation of different mouse tissues [187]. These advances will provide researchers and clinicians with deeper insights into HIV-1 biology and how it is modulated during replication in the different target cells, opening the field to a whole new world of therapeutic host targets to be explored during both active replication and reactivation from latency.

## Data Availability

Data are contained within the article.
